# Molecular mechanisms of neurite regeneration and repair: insights from *C. elegans* and *Drosophila*

**DOI:** 10.1186/s13619-022-00155-2

**Published:** 2023-04-02

**Authors:** Xiaofan Liu, Yuqing Zhao, Wei Zou

**Affiliations:** 1 The Fourth Affiliated Hospital, Zhejiang University School of Medicine, Yiwu, China; 2 Institute of Translational Medicine, Zhejiang University, Hangzhou, China; 3 Institute of Zhejiang University and University of Edinburgh, Jiaxing, China

**Keywords:** Neurite regeneration, Neurite repair, *Caenorhabditis elegans*, *Drosophila melanogaster*, Genetic manipulation, Live imaging, Subcellular structures, Axotomy, Dendrotomy, DLK-1

## Abstract

The difficulties of injured and degenerated neurons to regenerate neurites and regain functions are more significant than in other body tissues, making neurodegenerative and related diseases hard to cure. Uncovering the secrets of neural regeneration and how this process may be inhibited after injury will provide insights into novel management and potential treatments for these diseases. *Caenorhabditis elegans* and *Drosophila melanogaster* are two of the most widely used and well-established model organisms endowed with advantages in genetic manipulation and live imaging to explore this fundamental question about neural regeneration. Here, we review the classical models and techniques, and the involvement and cooperation of subcellular structures during neurite regeneration using these two organisms. Finally, we list several important open questions that we look forward to inspiring future research.

## Background

Axons play a key role in transmitting nerve impulses generated by neuronal cell bodies to other neurons or effectors. Dendrites are mainly responsible for receiving impulses from other neurons and transmitting them to the cell body. When subjected to injury such as cutting, toxic insults, etc., the neurite breaks down, causing functional impairment. Regeneration of proximal axons or dendrites is regulated by in vivo mechanisms. The regenerated neurite is expected to restore its original function. Therefore, neuronal regeneration mechanisms are essential for the maintenance of neuronal homeostasis.

As model animals, *C. elegans* and *Drosophila* have the advantages of clear genetic background, many progenies, and easy cultivation in the laboratory, which facilitates the study of neurite regeneration. Motor neurons and mechanosensory neurons of *C. elegans* are often used in the study of regeneration. For motor neurons, regeneration is usually assessed as the proportion of growth cones formed within 24 h the proportion of neurons reaching their original location within 24 h, and the time required to form growth cones (Hammarlund et al. 2009). For mechanosensory neurons, regeneration is often assessed as the length of neurites during regeneration (Wu et al. 2007). Dendritic arborization (da) neurons are often used as models of dendrite regeneration as sensory neurons in *Drosophila* because of their location that facilitates laser axotomy (Sugimura et al. 2003).

Subcellular structures such as organelles, as essential executors of cell functions, provide guarantees for the normal operation of cells. Subcellular structures such as cytoskeletal components, mitochondria, autophagosomes, endosomes, endoplasmic reticulum, nucleus, ribosomes and extracellular matrix, all play important roles in *C. elegans* and *Drosophila* regeneration. In the RNAi-based screen in *unc-70*/ *β*-spectrin mutants for defective motor axon regeneration in *C. elegans*, at least 50 conserved genes with growth-promoting or inhibiting functions were identified (Nix et al. 2014). p38 and JNK family MAP kinases associated with cytoskeleton dynamics have been shown to play critical roles in triggering injury signaling (Nix et al. 2011; Xiong et al. 2010). The dual-leucine zipper kinase 1 (DLK-1) is the most studied cellular intrinsic factor for axon regeneration. The DLK-1 pathway is essential for regeneration of motor neurons in *Caenorhabditis elegans*. Eliminating the DLK-1 pathway inhibits regeneration, while activating it promotes regeneration (Hammarlund et al. 2009). The notion that the DLK-1 cascade can be activated by cytoskeleton disruption independent of calcium elevation has been confirmed using *Drosophila* and mammalian sensory neurons (Valakh et al. 2015; Valakh et al. 2013). Activation of JNK-1 MAP kinase inhibits GABA neuronal regeneration. Notch and Wnt signaling pathways activated during development have also been implicated in axon regeneration (Bejjani and Hammarlund 2012). In addition, the conserved Arf Guanine-nucleotide Exchange Factor (GEF) EFA-6 was found to inhibit axon regrowth after laser surgery when using a laser injury model to screen mechanosensory neurons in *C. elegans* (Chen et al. 2011). In the laser injury model screening, it was also found that NMAT-2 uses its enzymatic activity to inhibit axon regeneration (Kim et al. 2018). Large-scale unbiased genetic screens for axon regeneration have not been performed in *Drosophila*. However, in the screening of genes regulated after axotomy, it was found that both Rtca and Archease, components of RNA repair and splicing pathways, have regulatory effects on axon regeneration (Song et al. 2015). The PTEN-Akt pathway is important for both axonal and dendritic regeneration in *Drosophila*. Nevertheless, the different requirements for JNK signaling allow the mechanisms of *Drosophila* dendritic regeneration to be distinguished from axon regeneration (Brace and DiAntonio 2017; Stone et al. 2014).

**Fig. 1:**
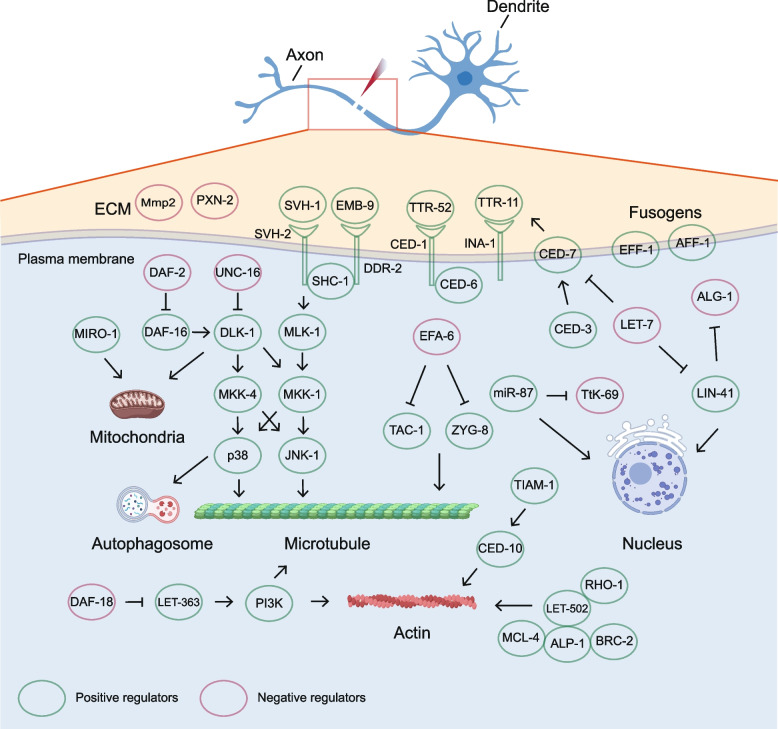
Regulators of neurite regeneration and associated subcellular structures in *C. elegans* and *Drosophila*. In red: negative regulators; In green: positive regulators

This review summarizes the relevant mechanisms and recent progress of neurite regeneration. We illustrate the advantages of *C. elegans* and *Drosophila* as commonly used model organisms to study neurite regeneration. Laser surgery, as a standard method for cutting neurons of worms and *Drosophila*, provides convenience for the study of neurite regeneration. Subcellular structures such as organelles play a non-negligible role in regenerating axons and dendrites (Fig. 1).

## Models and technologies

### *Caenorhabditis elegans*

*C. elegans* is a well-established animal model for basic neurobiology study, with significant advantages. Fundamentally, its short-life cycle, small and transparent body have greatly accelerated and facilitated scientific studies. At the genetic and molecular level, *C. elegans* is the first multicellular eukaryotic organism whose genome has been fully sequenced, and both its genome and major molecular pathways are highly conserved with mammals. At the cellular level, the number and composition of *C. elegans* hermaphrodite are invariable within individuals, including 302 well-characterized neurons with diverse functions and morphologies (White et al. 1986). These characteristics have made *C. elegans* an ideal model for investigating molecular and cellular mechanisms of neurite regeneration. During the past decades of intensive study, two categories of neurons were set up as models for axon and dendrite regeneration, respectively.

Compared with dendritic regeneration, axonal regeneration has a more extended research history and deeper understanding. Two types of neurons, GABA motor neuron and mechanosensory neuron, are used most frequently in this field. Specifically, 26 out of 302 neurons of the *C. elegans* nerve system are identified as GABA motor neurons, further characterized into three subtypes according to their synaptic outputs (Schuske et al. 2004). Among them, the dorsal- and ventral-innervated D-type motor neurons, which include six DD neurons and 13 VD neurons, are intensively used in axon regeneration studies (White et al. 1986). Morphologically, the cell bodies of these neurons are aligned regularly along the ventral nerve cord, and a branch of their anterior axons forms commissures that run towards the dorsal nerve cord. The axons of fluorescent-labelled D-type motor neurons are apparent under a microscope (Bejjani and Hammarlund 2012), making it a convenient target for axon injury like axotomy. Functionally, these neurons regulate the sinusoidal body movement of the nematode by relaxing the dorsal muscles when the ventral muscles are contracting. Hence, aside from directly observing the morphology of the neurons after axon injury, the coordinated movement of the animal can be seen as a reliable indicator of regeneration level.

The mechanosensory neuron (e.g., ALM and PLM) exhibits a longer axon than the GABA motor neuron, extending along the anterior–posterior axis. These characteristics have made the neurons another befitting model for axon injury and regeneration, and in a long distance and time scale compared with the GABA motor neurons. Comparing and contrasting the two types of neurons can diversify our understanding of axon regeneration and inspire ideas to investigate the more heterogeneous mammalian nervous system. Further, the function of the touch receptor neurons may also be reflected in the corresponding locomotion of *C. elegans* (i.e., the gentle touch response), through the quantitative experiment design and result analysis is much more complicated than it in the motor neurons (Vayndorf et al. 2016).

Dendritic regeneration is an emerging research hotspot, and the PVD neuron draws the most attention in this field for its delicate structured dendritic trees. A typical PVD neuron has four degrees of dendrites. Its primary dendrite runs along the A-P axis from head to tail, and the secondary, tertiary, quaternary branches appear perpendicular to each other to form multiple candelabra-like units (Oren-Suissa et al. 2010). Its unique and highly organized dendrites made it an ideal object for studying dendrite development, organization, maintenance, and regeneration. Like axotomy, dendrotomy can be applied to the PVD neuron for investigating multi aspects of dendrite regeneration: including the re-elongation and the re-arborization and recovery of its function (Brar et al. 2022; Oren-Suissa et al. 2017).

### *Drosophila melanogaster*

*Drosophila* is another powerful animal model used in research of neurite repair, and its dendritic arborization (da) sensory neuron is a favored injury model for both axon and dendrite. Generally, the *Drosophila* da neuron can be classified into four classes (class I-IV) based on the complexity of their dendrite pattern and can be further divided into several subgroups according to their location (e.g., dorsal, ventral). Noticeably, the regeneration after neurite injury exhibits different abilities and mechanisms among subgroups and between the central and peripheral nervous systems (CNS and PNS) (Rao and Rolls 2017; Vargas et al. 2020). These distinctions can specifically give inspiration for our understanding of the more complex nerve system in humans.

Class I, III, and IV da neurons are frequently used models for axon injury and repair study, and the axotomy using a two-photon laser is a well-established laboratory technique (Sugimura et al. 2003). The response of the severed axon can be classified into three situations: regenerated, stalled, and degenerated, and can be further quantified using a widely-accepted method. For example, the “regeneration index” is calculated by the increase of axon length divided by the distance between the cell body and the axon converging point, in which the regeneration length (or degeneration length) is normalized and better for comparison (Song et al. 2015).

Class IV da neuron (c4da), for example, the dorsal da C neuron (ddaC), is also an emerging model used for dendrite repair study of its elaborate structured dendrite tree. Another attractive characteristic of the c4da neuron is its spontaneous pruning and regrowth during metamorphosis. Although this development-induced regrowth and the injury-induced regeneration of dendrites have shared molecular effectors and pathways, their initiation, detailed mechanisms and outcomes are mainly distinctive. To be precise and specific, this article will not cover the regrowth process of neurites during development. Here, we summarize the characteristics and functions of different types of neurons during neural regeneration in *C. elegans* and *Drosophila* (Table 1).

**Table 1:** Types of neurons used for studying regeneration and repair in *C. elegans* and *Drosophila*

Subtype	Function	Research focus	Reference
C.*elegans* VD and DD neuron	Motor neuron; regulation of the sinusoidal body movement	Axon regeneration	Schuske et al. 2004
*C. elegans* DA9	Motor neuron; receive signals from neurons such as AVA and transmit to VD neurons	Axon regeneration	Ding and Hammarlund 2018
*C. elegans* ALM and PLM neuron	Sensory neuron; response to gentle touch to the different part of body	Axon regeneration	Chen et al. 2011
*C. elegans* ASJ neuron	Sensory neuron; control nematodes in and out of the dauer stage, sensing light and electricity	Axon regeneration	Chung et al. 2016
*C. elegans* PVD neuron	Sensory neuron; response to fast and high-displacement mechanical and aversive thermal stimuli	Dendrite regeneration; axon regeneration	Brar et al. 2022; Oren-Suissa et al. 2017; Zhao et al. 2021
*Drosophila* Motor neurons	Motor neurons; dominant muscle group	Axon regeneration	Xiong et al. 2010
*Drosophila* Class IV DA neurons	Sensory neuron; encode sensory input	Axon regeneration; dendrite regeneration	Song et al. 2019; Thompson-Peer et al. 2016
*Drosophila* Class I, III DA neurons	Sensory neuron; encode sensory input	Axon regeneration	Song et al. 2019

### Technologies

Femtosecond laser surgery is a typical method to cause injury in a single targeted axon or dendrite branch and has developed for decades since the early 2000s (Yanik et al. 2004). The high-energy pulse can vaporize the targeted process of a neuron in *C. elegans*, and with the aid of two-photon technology, the surgery can be carried out in larger animals like *Drosophila* and zebrafish (Galbraith and Terasaki 2003; Sugimura et al. 2003). In *Drosophila*, the Dumostar number 5 forceps can be used to pinch neurons through the cuticle (Xiong et al. 2010). Regeneration and repair mechanisms can be activated after the laser injury in the model neurons mentioned before, thus providing an ideal platform for studying injury-induced neurite regeneration pathways. Recently, a novel method with higher operability to cause dendrite injury was developed (Zhao et al. 2021). With a single microinjection needle, the dendrite of *C. elegans* PVD neurons can be truncated under the dissecting stereomicroscope rapidly. Here, we summarize techniques used to damage neurons to study neurite regeneration in *C. elegans* and *Drosophila* (Table 2).

**Table 2:** Comparison of technologies and their applications in studies of neurite regeneration and repair

Technologies	Application	Applicable model animal	Advantages	Disadvantages	References
Femtosecond laser surgery	Vaporizing targeted single axon or dendrite branch	*C. elegans*; *Drosophila*	Quick, and precise	Relatively expensive	Galbraith and Terasaki 2003; Sugimura et al. 2003; Yanik et al. 2004
Microinjection needle surgery	Mechanical truncation of single dendrite branch	*C. elegans*	Low cost and convenient	Requires intense training	Zhao et al. 2021
Forceps pinched surgery	Mechanical crushing of neuronal axons	*Drosophila*	Quick, low cost	Technically challenging	Xiong et al. 2010
Mechanical stress induced spontaneous axonal break	*unc-70/β*-spectrin mutants are capable of regenerating and repairing axonal breaks	*C. elegans*	Convenient	Only applies to specific neurons	Nix et al. 2014

Notably, both worms and flies are powerful genetic models with intensively investigated genomes. Genome-wide RNA interference (RNAi) libraries for *C. elegans* and *Drosophila* were set up early this century (Dietzl et al. 2007; Kamath 2003), enabling the high-resolution reverse genetic screening in identifying the key players in neurite regeneration. Recent advances in the CRISPR/Cas9 systems also enable high-efficient and customized techniques for time- and region-specific activation or inactivation of interested genes (Dickinson et al. 2013; Shen et al. 2014). Hence, together with diverse genetic screen methods and efficient neurite injury technologies, the precise roles of multiple genes and proteins during neurite regeneration processes can be identified. Below we will discuss several essential neurite regeneration pathways identified using these models.

## Neurite regeneration regulated by the subcellular structures

### Cytoskeletal components

The cytoskeletal components are fundamental components of all eukaryotic cells, composed of three elements: microtubule, microfilament, and intermediate filament. Take the microtubule for example; under physiological conditions, the microtubule consistently undergoes polymerization dynamics: a prolonged phase transition between polymerization and depolymerization (Desai and Mitchison 1997). Specifically, the two processes are highly regulated by many regulators, which can be divided into two groups: catastrophe factors that promote depolymerization and rescue factors that help to stabilize microtubules (Chen et al. 2018). Because of this character, the cytoskeletal components can provide a stable structure and dynamic movements and transport systems of a cell in response to different cellular needs, thus becoming an indispensable participant in multiple cellular processes, including neurite regeneration.

Immediately after injury, the cytoskeletal components are among the first lines of subcellular structures to be influenced. In *C. elegans* touch receptor neurons, most of the growing events of the microtubule proximal to the injury site halted within seconds (Chen et al. 2015). Another injury signal intensifies this destabilization; the elevation of intracellular calcium that forms a signal wave quickly spreads from the injury site to the distal part of the neuron (Ghosh-Roy et al. 2010). Calcium destabilizes microtubules by weakening the tubulin-tubulin interaction and can even result in the transition from stable axon shaft to growth cone (Weisenberg and Deery 1981; Ziv and Spira 1997). Consequently, the calcium wave and the disrupted dynamic balance of the microtubule both act as a signal to trigger several downstream injury response factors.

As a MAP kinase kinase kinase, DLK-1 acts upstream of the p38 and JNK families of the mitogen-activated protein kinase (MAPK) pathway (Hirai et al. 1996, Nix et al. 2011). Also, as a highly conserved pathway, studies in multiple model organisms, from worms to *Drosophila* and even mammals, have revealed its role as a central regulator in the axon regeneration process (Summers et al. 2020; Yan et al. 2009). Microtubule dynamics regulation is one of its downstream effects. In *Drosophila*, regulation of microtubule dynamics and polarity by the JNK pathway can initiate neurite regeneration and even regenerate an axon from dendrite (Stone et al. 2010). In *C. elegans*, loss of DLK-1 leads to inhibition of axonal regrowth after injury, while its overexpression can promote regeneration by inhibiting a microtubule catastrophe factor, kinesin-13 (Ghosh-Roy et al. 2012). Recent advances using *C. elegans* also identified UNC-16 (the homologue of the vertebrate JIP-3), which acts as an inhibitor of the DLK-1 pathway after axon injury by altering the availability of the functional DLK-1 protein rather than downregulating its kinase activity at the injured site (Kulkarni et al. 2021). As a result, the dynamics of the microtubule and the building block of microfilament, actin, are suppressed, and axonal regrowth is inhibited. However, in the nematode amphibious neuron ASJ, severing dendrites enhanced DLK-1-independent axonal regeneration. This axonal regeneration is dependent on the transduction of calcium signaling through the ion channel (Chung et al. 2016). In addition, PVD dendritic regeneration in *C. elegans* is not dependent on the DLK pathway, but the TIAM-1/CED-10 cascade cells autonomously initiate dendritic regeneration followed by branching (Brar et al. 2022).

In contrast to DLK-1, EFA-6 (the guanine-nucleotide exchange factor for ARF-6) is viewed as a catastrophe factor for cytoskeleton dynamics after injury (Chen et al. 2015). Its inhibitory effects on axon regeneration are first identified in *C. elegans* mechanosensory neurons by a systemic genetic screen (Chen et al. 2011). Particularly, EFA-6 quickly re-localizes from the cell membrane to the minus ends of the microtubule and downregulates axonal microtubule dynamic via its interaction with microtubule-associated protein, TAC-1 and ZYG-8 (Chen et al. 2015). EFA-6 activates ARF-6 upon neurite injury, resulting in integrin’s retrograde transportation and inhibited regeneration (Chen et al. 2015). In *Drosophila*, Efa6 also acts as a regulator of axon growth by suppressing the microtubule polymerization during development (Qu et al. 2019). The inhibition effects of EFA-6 do not last long after injury in some cases; instead, the protein may re-localize back to the plasma membrane, and the microtubule dynamics will upregulate consequently (Chen et al. 2015).

Besides the two major pathways regulating cytoskeleton dynamics after neurite injury, other molecules also participate in the process. For instance, the MLK-1 cascade can upregulate the growth cone formation in *C. elegans* GABA motor neuron after axotomy in cooperation with the DLK-1 pathway (Nix et al. 2011). BRC-2 and ALP-1 in *C. elegans* positively regulate the RHO-1/Rho GTPase- LET-502/ROCK pathway then promote MLC-4 phosphorylation, thereby promoting axonal regeneration (Shimizu et al. 2018). In aged *C. elegans*, the insulin signaling pathway DAF-2/INSR/IGF1R inhibits the formation of regenerative growth cone by sequestering the forkhead transcription factor DAF-16/FOXO (Byrne et al. 2014). Independent of the insulin cascade, another highly conserved pathway, the PTEN/mTOR pathway, also influences cytoskeleton dynamics during axon regeneration. Inactivation of PTEN can disinhibit the mTOR pathway and trigger some of its downstream regulators such as PI3K and GSK-3 to enhance axonal transport and cytoskeleton assembly (Park et al. 2010). In mice, mTOR is important for insulin-mediated RGC dendrite regeneration. mTOR complex 1 (mTORC1) is required to restore dendritic complexity, while mTORC2 is required to restore dendritic coverage (Agostinone et al. 2018).

### Mitochondria and endoplasmic reticulum

Mitochondria are vital organelles responsible for energy metabolism, signal transduction, and calcium homeostasis. In normal axons, mitochondria are transported to the plus and minus ends along microtubule filaments by the motor proteins kinesin and dynein, respectively (Lin and Sheng 2015).

After the axons of GABA motor neurons of *C. elegans* are injured, mitochondria will translocate into the axons, increasing the mitochondrial density in the axons. Mitochondrial translocation is regulated by double-leucine zipper kinase 1 (DLK-1). Mitochondria in axons are required for growth cones. After knocking down the *miro-1* gene responsible for mitochondrial transport, the overall length of axon regeneration was shortened (Han et al. 2016). In the absence of increased mitochondria in the injured axon, the axon is less able to regenerate (Han et al. 2016). In mice, the axon-specific mitochondrial outer membrane protein syntaphilin (SNPH) inhibits motility by increasing the force between mitochondria and microtubules and inhibiting ATPase activity (Chen and Sheng 2013). In SNPH knockout mice, mitochondrial trafficking is enhanced, ultimately reversing the energy deficit in axon regeneration (Cheng and Sheng 2021; Zhou et al. 2016). When *Drp1* conditional knock-out mice suffered axonal injury, mitochondrial morphology was altered with more long mitochondria, at which time acute and transient mitochondrial fission activation was important for maintaining neuronal and mitochondrial integrity (Kiryu-Seo et al. 2016). Therefore, recruitment of mitochondria onto the injury sites of axons may be one of the conserved ways to promote axon regeneration.

As a membrane organelle, the endoplasmic reticulum (ER) plays an important role in lipid synthesis, membrane protein synthesis and distribution, and calcium homeostasis. The structural morphology of the ER is highly dynamic and can well meet changing cellular demands during neuronal regeneration (Petrova et al. 2021).

In *Drosophila* ddaE neurons, the smooth ER is distributed throughout neuronal axons. After ddaE axonal injury, hereditary spastic paraplegia (HSP) protein and smooth ER accumulate at the tips of regenerating axons, but this was not observed in dendrites. Consistently, ER accumulation at the tips of regenerating axons was not seen in spastin null/hypomorph and atlastin RNAi animals (Rao et al. 2016). In mammals, Protrudin is an integral endoplasmic reticulum membrane protein that promotes regeneration by acting as a scaffold connecting axonal organelles, motors, and membranes (Petrova et al. 2020).

The linker molecules of mitochondria and ER also play a role in regeneration. Glucose regulated protein 75 (Grp75) accumulates in injured axons as a protein linking ER to mitochondria in mice. Overexpression of Grp75 can produce more mitochondrial calcium and ATP, and promote axon regeneration (Lee et al. 2019).

### Autophagosomes

Autophagy is a process that degrades unwanted or dysfunctional organelles and proteins in an autophagosome-lysosome-dependent manner, thus being crucial for cellular homeostasis (Levine and Klionsky 2004). Upon neurite injury, timely removal of debris near the injury site is critical for the following regeneration events. Hence, the regulation of autophagy is another vital aspect of neurite regeneration.

In a recent study using *C. elegans* as a model organism, both autophagosomes and autolysosomes are reported to elevate after axotomy in an age-dependent manner (Ko et al. 2020). The injury-induced autophagy is dependent on the DLK-1 signaling pathway and can limit the function of NOTCH protein, an inhibitor for axon repair (Ko et al. 2020). In mice, local administration of an autophagy-induced peptide to the axonal injury site significantly suppresses axonal retraction and improves the neuron recovery by stabilizing the microtubule structure (He et al. 2016). The inhibition of intracellular mTOR signaling pathway via rapamycin administration forms the molecular underpinnings of the blockade of Schwann cell-mediated autophagy, which accelerates the axon regeneration of peripheral nerves in mammalians (Inada et al. 2021; Li et al. 2020).

### Nucleus and ribosomes

microRNA (miRNA) are small non-coding RNAs that play a vital role in the post-translational regulation of gene regulation, thus potentially influencing the neurite regeneration upstream of the previously mentioned key factors (Bushati and Cohen 2007).

In *C. elegans* AVM neuronal axons, a robust axon regeneration phenotype is shown when miRNA is depleted. In older neurons, *let-7* miRNA acts on the 3’ untranslated region of *lin-41,* an essential AVM axon regeneration promoting factor, to inhibit *lin-41* expression and suppress axon regeneration. In younger neurons, *lin-41* inhibits the expression of *let-7* through the Argonaute ALG-1, showing a phenotype of axon regeneration (Zou et al. 2013). Loss of *let-7* can also enhance axon regeneration by increasing the expression of *ced-7*, and, consequently, disinhibit the EFF-1–mediated axonal self-fusion (Basu et al. 2017). In the dendrites of *Drosophila* C4da neurons, however, the miRNA does just the opposite. The important regeneration regulator miRNA miR-87 promotes regeneration by inhibiting the transcriptional repressor Tramtrack69 (Ttk69). When miR-87 is absent, pruned dendrite regeneration is impaired. However, overexpression of miR-87 resulted in premature initiation of dendritic regeneration (Kitatani et al. 2020).

### Plasma membranes and extracellular components

#### Fusogen-mediated plasma membrane fusion

When neurons are damaged, the damaged axons or dendrites need to reconnect with the original tissue. The fusion of neurons becomes the mechanism of possible spontaneous regeneration that reconnects the target. In *C. elegans*, when PLM neuron axons are severed, the fusogen protein EFF-1 is originally distributed throughout the PLM neurons to appear on the membrane at the tips of the proximal and distal segments of the cut site. When phosphatidylserine (PS) is exposed to damaged neurons as a 'save-me' signal, conserved apoptotic cell clearance molecules such as transthyretin TTR-52 respond. Then downstream EFF-1 restores membrane and cytoplasmic continuity, enabling axon regrowth and reconnection (Neumann et al. 2015). Specifically, another transthyretin-like protein, TTR-11, act as a mediator of injured axon recognition by cross-linking with the PS and integrin and initiating the axonal regeneration process (Hisamoto et al. 2018). EFF-1-related transmembrane protein AFF-1 mediates dendrite regeneration when PVD neuron dendrites are severed. The fusogen protein AFF-1 functions in cell–cell fusion in the skin and reproductive systems. Extracellular vesicles containing AFF-1 are released from the seam cells, promoting dendrite fusion when PVD dendrites are damaged (Oren-Suissa et al. 2017). When AFF-1 is overexpressed in PVD, the proportion of dendritic fusion regeneration in aged animals is significantly higher than that in wild type. Therefore, AFF-1 can regulate regeneration after dendritic injury (Kravtsov et al. 2017).

#### The extracellular matrix

The extracellular matrix (ECM) plays an essential role in the stability of the neuronal structure, synaptogenesis and neurite regeneration. Neurons and glial cells synthesize chondroitin sulfate proteoglycans (CSPGs), tenascin-R and hyaluronic acid to form the ECM. CSPGs-degrading matrix metalloproteinases (MMPs) may promote axon regeneration in the context of axonal injury (Cua et al. 2013). In *Drosophila* C4da dendrites, regeneration is increased when matrix metalloproteinase 2 (Mmp2) is inactivated to inhibit ECM degradation (DeVault et al. 2018). Many axons in *C. elegans* grow between the epidermis and the body wall muscles, relying on the basement membrane for their proper attachment. The peroxidase PXN-2 plays a vital role in basement membrane consolidation. Loss of *pxn-2* can promote the regeneration of damaged PLM and ALM neurons (Gotenstein et al. 2010).

#### Secretory vesicles

Secretory vesicles are generated from the Golgi network, undergo maturation, and translocate to their target plasma membrane. The trafficking of secretory vesicles is thought to be regulated by Rab small GTPases, such as Rab3 and its relatives involved in regulating exocytosis (Gabriele Fischer von Mollard et al. 1994).

In *C. elegans*, knockdown of the Rab GTPase *rab-27* resulted in an enhanced regenerative phenotype of GABA neurons (Sekine et al. 2018). In a recent study, RAB-27-dependent neuropeptide NLP-40 was found to be secreted from the intestine to inhibit regeneration of injured GABA neurons (Lin-Moore et al. 2021).

#### Other external factors

Axon regeneration is also regulated by external factors. SVH-1 is secreted from ADL sensory neurons and activates the receptor-type tyrosine kinase SVH-2 in injured D-type motor neurons, thereby regulating the MLK-1–MEK-1–KGB-1 JNK pathway to promote regeneration of D-type motor neuron (Hisamoto et al. 2016, Li et al. 2012). As a transmembrane collagen-binding RTK, DDR-2 can co-regulate axonal regeneration with EMB-9 collagen type IV. When DDR-2 was knocked out, the frequency of axon regeneration in worms decreased. Scaffolding protein SHC-1 can simultaneously interact with DDR-2 and SVH-2 to regulate axon regeneration (Hisamoto et al. 2016). Recent advances found the participation of other distal tissues in inhibiting the regeneration of axons. Under normal circumstances, the mature central nervous system of *Drosophila* is almost unable to regenerate after injury. Nevertheless, glial cells can be reprogrammed to increase glycolysis, thereby producing more metabolites L-lactate and L-2-hydroxyglutarate, and promoting axon regeneration in C4da neurons (Li et al. 2020).

## Conclusions and perspectives

Studies using *C. elegans* and *Drosophila* to investigate the mechanism of neurite regeneration have yielded exciting advancements, including the identification of several organelles and conserved pathways (Table 3). However, we are still far from fully seeing the entire story.

**Table 3:** Regulators during neural regeneration in *C. elegans* and *Drosophila* and their mammalian homologues

Invertebrate	Vertebrate	Regulator	References
DLK-1	MAP3K12	Activator	Ghosh-Roy et al. 2012; Hirai et al. 1996; Nix et al. 2011
JNK-1	MAPK10, MAPK8	Activator	Stone et al. 2010
UNC-16	MAPK8IP3, SPAG9	Inhibitor	Kulkarni et al. 2021
EFA-6	PSD3	Inhibitor	Chen et al. 2015
TAC-1	TAC1	Activator	Chen et al. 2015
ZYG-8	DCLK1	Activator	Chen et al. 2015
MLK-1	MAP3K9	Activator	Nix et al. 2011
BRC-2	BRCA2	Activator	Shimizu et al. 2018
ALP-1	LDB3; PDLIM5; PDLIM7	Activator	Shimizu et al. 2018
DAF-2	IGF1R	Inhibitor	Byrne et al. 2014
DAF-16	FOXO	Activator	Byrne et al. 2014
DAF-18	PTEN	Inhibitor	Byrne et al. 2014
LET-363	TOR	Activator	Byrne et al. 2014
MIRO-1	MIRO	Activator	Han et al. 2016
*let-7*	/	Inhibitor	Zou et al. 2013
LIN-41	TRIM71	Activator	Zou et al. 2013
ALG-1	AGO2	Inhibitor	Zou et al. 2013
miR-87	/	Activator	Kitatani et al. 2020
Ttk69	/	Inhibitor	Kitatani et al. 2020
CED-7	ABCA3	Activator	Basu et al. 2017; Neumann et al. 2015
TTR-52	/	Activator	Neumann et al. 2015
TTR-11	/	Activator	Hisamoto et al. 2018
EFF-1	/	Activator	Basu et al. 2017; Neumann et al. 2015
AFF-1	/	Activator	Oren-Suissa et al. 2017
Mmp2	MMP14; MMP15; MMP16	Inhibitor	DeVault et al. 2018
PXN-2	PXDN	Inhibitor	Gotenstein et al. 2010
AEX-6	RAB27A	Inhibitor	Lin-Moore et al. 2021
SVH-1	TMPRSS15	Activator	Li et al. 2012
SVH-2	TYRO3	Activator	Hisamoto et al. 2016; Li et al. 2012
DDR-2	DDR1; DDR2	Activator	Hisamoto et al. 2016
SHC-1	SHC1	Activator	Hisamoto et al. 2016

Although the intensive investigation of some organelles and subcellular structures, such as the cytoskeleton and plasma membrane, our understanding of other participants in the neurite regeneration process is still relatively young. The metabolism regulation by mitochondria, autophagy process mediated by autophagic vesicles, and involvement of extracellular components are all promising research directions in this field. Moreover, the interaction between these organelles is still poorly understood. For instance, the mTOR pathway, which is believed to promote neurite regeneration by enhancing cytoskeleton dynamics, also negatively affects regeneration by inhibiting injury-induced autophagy. Hence, understanding the involvement of subcellular structures in an integrated manner may greatly expand our knowledge of neurite regeneration.

Also, profit from the proven genetic screen and manipulation techniques developed for the two model organisms, some of the novel genetic clues may still hide in the dataset, waiting for further investigation. We also need to pay extra attention to the normal function of regenerated neurites, for some studies raised the concerns that partially impaired function and abnormal morphology of neurites despite complete regeneration after injury (Ding and Hammarlund 2018; Thompson-Peer et al. 2016).

Like doing jigsaw puzzles, once we have collected almost all the scattered pieces, we can explore the interactions between these involved organelles and pathways, gradually construct the general framework of the neurite regeneration process and potentially identify the most influential factors of the whole story and how they can be organized and manipulated in an integrative manner to reach higher efficiency of neurite regeneration. Noticeably, the manipulation of regeneration-related genes should be done with great care, for many of them have other roles in physical conditions. For example, a gene that suppresses axon regeneration may also be crucial in suppressing ectopic neurite sprouting during development. The compensation effects of gene editing can also give rise to unwanted side effects.

By extending the findings derived from *C. elegans* and *Drosophila* to other species, and finally, the mammals, we can reach a final goal of reversing the degeneration and injured neurons and curing these currently incurable diseases.

## Data Availability

Not applicable.

## Abbreviations

*C. elegans*: *Caenorhabditis elegans*
da neuron: Dendrite arborization neuron
GEF: Guanine-nucleotide Exchange Factor
ALM: Anterior lateral microtubule cells
PLM: Posterior lateral microtubule cells
A-P axis: Anterior-posterior axis
CNS: Central nervous system
PNS: Peripheral nervous system
c4da: Class IV dendrite arborization neuron
ddaC: Dorsal dendrite arborization neuron
RNAi: RNA interference
CRISPR: Clustered regularly interspaced short palindromic repeats
Cas9: CRISPR associated protein 9
PTEN: Phosphatase and tensin homolog
mTOR: The mechanistic target of rapamycin
PI3K: Phosphoinositide 3-kinase
SNPH: Syntaphilin
ER: Endoplasmic reticulum
ATP: Adenosine triphosphate
miRNA: MicroRNA
PS: Phosphatidylserine
ECM: Extracellular matrix
